# Analysis of the Viscoelastic Properties of the Human Cornea Using Scheimpflug Imaging in Inflation Experiment of Eye Globes

**DOI:** 10.1371/journal.pone.0112169

**Published:** 2014-11-14

**Authors:** Giuseppe Lombardo, Sebastiano Serrao, Marianna Rosati, Marco Lombardo

**Affiliations:** 1 CNR-IPCF, Unit of Support of Cosenza, Ponte P. Bucci, 87036 Rende, Italy; 2 Vision Engineering Italy S.r.l., Via Adda 7, 00198 Rome, Italy; 3 Fondazione G.B. Bietti IRCCS, Via Livenza 3, 00198 Rome, Italy; University of Missouri-Columbia, United States of America

## Abstract

**Purpose:**

To demonstrate a Scheimpflug-based imaging procedure for investigating the depth- and time-dependent strain response of the human cornea to inflation testing of whole eye globes.

**Methods:**

Six specimens, three of which with intact corneal epithelium, were mounted in a customized apparatus within a humidity and temperature-monitored wet chamber. Each specimen was subjected to two mechanical tests in order to measure corneal strain resulting from application of cyclic (*cyclic* regimen) and constant (*creep* regimen) stress by changing the intra-ocular pressure (IOP) within physiological ranges (18–42 mmHg). Corneal shape changes were analyzed as a function of IOP and both corneal stress-strain curves and creep curves were generated.

**Results:**

The procedure was highly accurate and repeatable. Upon cyclic stress application, a biomechanical corneal elasticity gradient was found in the front-back direction. The average Young's modulus of the anterior cornea ranged between 2.28±0.87 MPa and 3.30±0.90 MPa in specimens with and without intact epithelium (P = 0.05) respectively. The Young's modulus of the posterior cornea was on average 0.21±0.09 MPa and 0.17±0.06 MPa (P>0.05) respectively. The time-dependent strain response of the cornea to *creep* testing was quantified by fitting data to a modified Zener model for extracting both the relaxation time and compliance function.

**Conclusion:**

C*yclic* and *creep* mechanical tests are valuable for investigating the strain response of the intact human cornea within physiological IOP ranges, providing meaningful results that can be translated to clinic. The presence of epithelium influences the results of anterior corneal shape changes when monitoring deformation via Scheimpflug imaging in inflation experiments of whole eye globes.

## Introduction

The corneal tissue is the main refractive element of the human eye. Understanding the corneal shape and thickness and their relation with the tissue's mechanical properties is important to refractive or therapeutic corneal treatments. Measurements of human corneal biomechanics traditionally are destructive and performed after tissue excision. On the other hand, the mechanical properties of the intact tissue are fundamentally related to the anterior and posterior corneal shapes.

Mapping corneal deformation non-invasively in response to variable intraocular pressure (IOP) changes can be highly valuable to understand the viscoelastic properties of the intact tissue in situ. In this context, corneal topographers permit rapid and detailed characterization of the cornea. Modeling the corneal topography changes caused by IOP loading and unloading of the whole eye globe, as done via inflation testing, represents one of the most appropriate procedures to investigate *ex vivo* the biomechanical properties of the corneal tissue. The use of the intact eye and measure of corneal shape changes by corneal topography provide closer situation to understand the natural behaviour of the cornea that can be readily translated to clinical practice [1], [2]. Different mathematical models [3]–[7] have been proposed to fit data measured by computerized topographers, which included the use of surface models like sphere, conic or biconic, with the last model providing most of the basic optical properties of the cornea.

In the present study, we show a procedure to evaluate the biomechanical properties of the human corneal tissue through the analysis of corneal shape changes via Scheimpflug imaging in inflation testing of whole eye globes. The procedure was developed to investigate the corneal shape changes both to *cyclic* stress variation and *creep* by changing IOP within physiological ranges (18–42 mmHg). Stress-strain curves obtained in loading/unloading (*cyclic*) testing provide information about elasticity and hysteresis of the tissue. *Creep* testing, by subjecting the tissue to a steady force and measuring the deformation over time, provides information about the time-dependent strain of the cornea. Clinically, corneal *creep* may be an important contributor to the pathogenesis of ectasia in keratoconus or post-laser keratectomy [8], [9]. To the best of our knowledge, this is the first work aiming at investigating the creep behaviour of the human cornea in inflation experiment of whole eye globes.

## Methods

Six human eye globes, from different donors, were obtained from the Veneto Eye Bank Foundation (Venezia Zelarino, Italy). All human eyes were used in compliance with the guidelines of the Declaration of Helsinki for research involving the use of human tissue and the experimental protocol was approved by the local ethical committee (Azienda Sanitaria Locale Roma A, Rome, Italy). The eye globes were explanted between 3 and 16 hours after death and immediately preserved at 4°C in corneal storage medium enriched with 15% dextran. Specimens were used for experiments within 48 hours.

### 2.1 Experimental apparatus

A custom-built apparatus, the Ocular Biomechanics Modulator (OBM), was used to perform experiments in a monitored environment. The schematic and picture of the OBM apparatus have been shown in previous work [1]. The temperature and humidity of the moist chamber, where the eye globe is placed during the experiment, was continuously monitored and recorded. The IOP of the eye globe was monitored by means of a water column and a pressure transducer (DS27C002A1, Valcom, Milano, Italy), and modified by infusing saline solution into the posterior segment of the eye through a needle by an automated pumping system (Genie Plus syringe pump, Kent Scientific Corp., CT, USA). Corneal topography maps of the anterior and posterior cornea were obtained using a Scheimpflug-based Pentacam HR (Oculus GmbH, Wetzlar, Germany). The whole apparatus was computer-controlled by dedicated software written in LabView (National Instruments Corporation, Austin, TX, USA).

### 2.2 Testing procedure

Six eye globes, of which three (50%) with intact epithelium and three (50%) that were de-epithelialized, were used for experiment. The specimens were allocated so as to have age-matched samples in the two groups. The de-epithelialized specimens were used in order to understand the contribution of epithelium to corneal shape changes in inflation testing of whole eye globes.

A set of control experiments was performed on three specimens in order to determine the testing protocol used in this work (data not shown).

**Figure 1:**
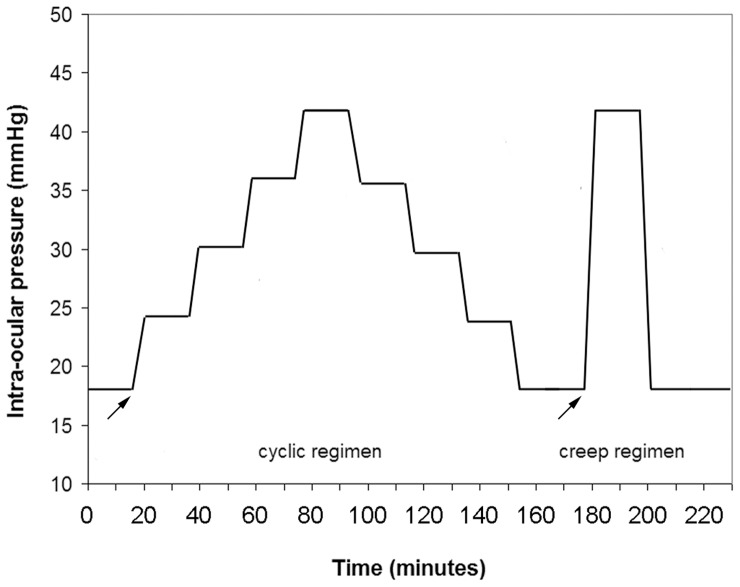
Stress-controlled procedure for *cyclic* and *creep* mechanical regimens. The two mechanical testing regimens (arrows) were preceded by a *pre-stressing* regimen (15 minutes) in order to attain an unique reference state between specimens. The *cyclic* regimen consisted in loading and unloading cycles; the *creep* regimen followed to *cyclic* regimen (i.e., 30 minutes after the last IOP change of the *cyclic* regimen) and consisted in a constant-stress hold.

Each specimen was kept in 15% dextran enriched storage solution at room temperature for 30 minutes before commencing the experiment. Thereafter, each eye globe was gently mounted into a specially designed holder, with known vertical/horizontal orientation, to guarantee proper centration during the topography measurements. The eye holder was placed in an acrylate box, where an ultrasound humidifier (JC 380, Life Tool Technologies, Ancona, Italy) provided a constantly humid atmosphere. A commercial air conditioner heat pump was used to maintain the room temperature constant. For each eye globe, a *cyclic* regimen followed by a *creep* regimen was applied. Both regimens started after the IOP was kept constant at 18 mmHg for 15 minutes (pre-stressing), as shown in Figure 1. The *cyclic* regimen consisted in an initial measurement taken at 18 mmHg and subsequently at 6 mmHg step increases, while continuously controlling the pressure trough the monitoring system. Pressure was increased to 42 mmHg and then decreased, at 6 mmHg steps, up to 18 mmHg. For each eye, Pentacam measurements were taken two times 10 minutes after each IOP change, as determined in control experiments where deformation recovered between each step. The *creep* regimen consisted in a nearly instantaneous IOP increase from 18 to 42 mmHg, then holding the IOP stable at 42 mmHg for 10 minutes while acquiring Pentacam images (twice) every 2.5 minutes. The IOP was then instantaneously decreased from 42 to 18 mmHg and held stable at 18 mmHg for 10 minutes while acquiring Pentacam images (twice) every 2.5 minutes.

### 2.3 Corneal model

We exported topographic maps of the anterior and posterior cornea from Pentacam and used technical computing software (Matlab, The MathWorks Inc., Natick, MA, USA) for data processing and analysis.

For precise reconstruction of corneal shape changes induced by inflation experiments and comparison of corneal maps, proper alignment of the anterior and posterior corneal interfaces is critical to prevent the misalignment artifacts from affecting the difference maps. Navarro et al. [6] showed a mathematical model to minimize misalignment, tilting and slanting of the cornea with respect to the measuring device. The alignment was performed mathematically using the elevation map measured by the topographer. This procedure provided corneal parameters depending only on the shape of the corneal surface and not on the relative cornea-instrument position, thereby correcting for misalignment errors.

Starting from the Navarro's model, we developed a mathematical procedure in order to unequivocally fit the elevation topographic data, i.e., the *x, y* and *z* points coordinates, with a non revolution general ellipsoid which is expressed as a second order degree polynomial in the form of:
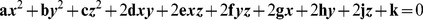
(1)where the parameters *a*, *b*, *c*, *d*, *e*, *f*, *g*, *h*, *j* and *k* are the unknowns variables. Considered that not all the quadratics are ellipsoids, it is therefore mandatory to find suitable constraints that those parameters must satisfy. The fit method used in this work is based on a two steps procedure by using a least squares criterion. As the equation (1) is non linear, before solving the nonlinear data-fitting problems in a least squares sense, it is essential to find an initial starting values for the vector of unknown parameters A, composed by [*a*, *b*, *c*, *d*, *e*, *f*, *g*, *h*, *j*] having normalized the equation (1) by the value of -*k*. Hence, the initial values for the unknown parameters are found by minimizing the cost function:
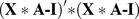
(2)with respect to *A*, where *X* is the matrix of size (*n*×9) formed by [x_i_^2^, y_i_^2^, z_i_^2^, 2x_i_y_i_, 2x_i_z_i_, 2y_i_z_i_, 2x_i_, 2y_i_, 2z_i_] where *n* are the numbers of the acquired elevation topographic data, and *I* is a vector of size (n×1) composed by 1 and the sign' is the transpose operator. Minimizing equation (2) with respect to *A* yields:
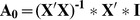
(3)where *A_0_* is the solution of the normal system of equation (3) and, since the matrix *X′X* has rank maximum (n = 9) and is symmetric and definite positive, *A_0_* is the **unique solution** which minimizes the squared 2-norm of the residual c = X*A-*I*.

Once obtained the general expression of the ellipsoid which best fits the topographic elevation data, one can extract the three orthogonal ellipsoid semiaxes which are able to fully describe the main corneal surface curvature features, such as the curvature radii and the asphericity constants associated with the two principal meridians of the cornea (i.e., the horizontal and vertical meridians). In other words, the most affine transformation as possible is found to transform the general model of the ellipsoid, expressed in the coordinate system of the topographer, to the main axes of symmetry of the cornea. By using standard linear algebra procedures, consisting of i) finding the center of the ellipsoid, ii) translating the ellipsoid with respect to its center, and iii) diagonalizing the resultant quadratic form in order to find its eigenvector and the associated eigenvalues, the ellipsoid can be then expressed in its canonical form:
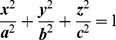
(4)where *a*, *b* and *c* are the semiaxes of the ellipsoid whose directions are aligned along the eigenvector and whose values are proportional to the associated eigenvalues. The final ellipsoid expression, used to compare the different ellipsoid model fitted with the elevation data, is translated along the optical axis of the corneal ellipsoid, i.e., the *z* direction, in order that the apical surface point is centered in (0, 0, 0).

The radii of curvature (mm) of the horizontal (R_x_) and vertical (R_y_) meridians of the anterior and posterior cornea were computed by fitting the central 6.00 mm of the corneal elevation maps to the model. The average changes of central corneal radii were plotted as a function of IOP. Thickness data were obtained directly from the Pentacam software.

### 2.4 Biomechanical models

The elastic modulus of the corneal tissue was estimated by using a model for the corneal stress-strain relationship based on IOP variation and related changes in radius of curvature and thickness, as previously shown [8], [10]–[13]. The model was based on assumption that changes of corneal thickness and radius of curvature are caused by IOP. Intraocular pressure applied to the posterior corneal surface was translated from radial stress to membrane stress with the Laplace law, as follows:
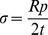
(5)where σ represents stress; *R* represents the mean radii [(R_x_+R_y_)/2] of anterior or posterior curvature; *p* represents current IOP; and *t* represents central corneal thickness. The stress-strain relationship from the IOP data was extracted using the following equation: ε = Δ*R*/*R_0_*; where ε represents strain, Δ*R* represents difference in radius of curvature with respect to the initial measurement, and *R_0_* represents initial radius of curvature. The model did not include potential meridional anisotropy behaviour in deformation; in other words, the cornea was assumed to remain spherical under pressure changes. The modulus of elasticity (Young's modulus, *E*) of the anterior and posterior cornea was calculated by plotting stress and strain values obtained during the loading phase of the *cyclic* regimen. The Young's modulus was estimated as the slope of the loading curve in the linear region. The relationship between regional elastic moduli and membrane stress has been validated in previous work [11] using a power model that fitted 99% of experimental data acquired in inflation experiments of whole eye globes.

**Figure 2:**
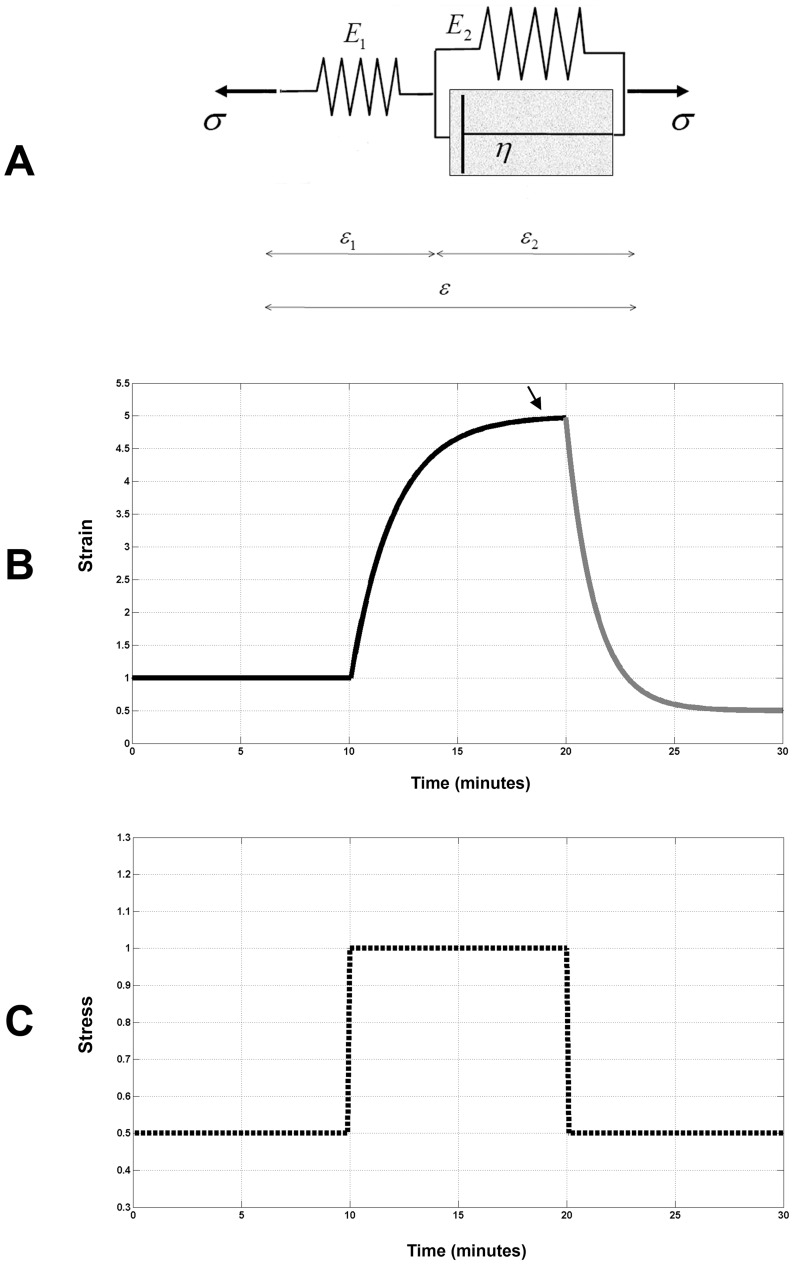
Modified Zener model. A) The Zener model consists of two elements represented by a spring in series with a Kelvin unit. The model was used (B) to approximate the strain response of the cornea (C) to constant stress application. Upon sudden loading (B, black curve), the first element of the model, represented by a spring with stiffness E_1_, stretches immediately. The dashpot of the second element, with viscosity η, then takes up the stress, transferring the load to the second spring, with stiffness E_2_, as it slowly opens over time. The strain (ε) should reach a steady constant value (arrow). Upon sudden unloading (B, grey curve), the spring E_1_ contracts immediately while the spring E_2_ slowly contracts, being held back by the dashpot. The full response predicted by the model is fairly close to the response of the corneal tissue to physiological IOP variation.

The time dependency of the anterior and posterior corneal strain to the application of a steady stress [14] was described by means of the creep compliance function derived by the Zener, or standard linear solid, model (Figure 2). The model has been previously shown to well approximate the viscoelastic response of the corneal tissue, whose conformational changes is limited by the network of junction points (e.g., stromal protein cross-linking bonds) [13]. It, however, cannot describe the permanent strain that may be left in the corneal tissue in a *creep* test. For that reason, we restated the creep compliance function in order to take into account the permanent strain and the relaxation times upon loading and unloading. In particular, the creep compliance function (Φ) was considered to consist of two main components, the unrelaxed compliance (***C_u_***) and the relaxed compliance (***C***_∞_), depending on whether the stress is applied (loading creep curve): 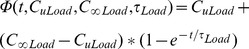
(6)or relaxed (unloading creep curve):
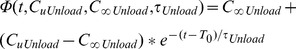
(7)where ***C_u_*** (either under loading or unloading) characterizes the immediate elastic response and ***C***_∞_ (either under loading or unloading) characterizes the steady state response, which includes the viscous flow, the retard elastic and the permanent strain responses of the model; these coefficients are proportional to the inverse of the stiffness E_1_ and E_2_ of the Zener model respectively. The coefficients *τ_load_* and *τ_unload_* are the relaxation times and represent a measure of how quickly the cornea relaxes to loading and unloading respectively; *t* is time; and *T*_0_ is the instant time at which the applied stress is removed. In this work, we used the ***C***_∞_ and *τ* parameters to quantify the strain response of the cornea to *creep* testing.

### 2.5 Statistics

The accuracy of the Pentacam instrument to evaluate the radius of curvature was determined by imaging a corneal model (acrylic material, LE-124, Eyetech Ltd., Morton Grove, Ill, USA) with known geometry. The experimental error was defined as the difference between a set of three repeated experimental measurements and the nominal values of the corneal model. The percent error, reported as a percentage, was used to determine accuracy of the instrument and was defined as the ratio of the error to the nominal value. The repeatability of Pentacam measurements was calculated based on the within-specimen standard deviation (σ_w_), that is the common standard deviation of repeated measurements. To get the common within-specimen standard deviation, we averaged the variances of the repeated measures in loading and unloading steps of *cyclic* regimen for each specimen. The repeatability was defined as 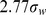 and reported both in terms of the measurement unit (mm or µm) as well as a percentage of the mean. The 95% confidence interval (CI) for repeatability was calculated as 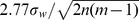, where *n* is the number of specimens and *m* is the number of measurements for each specimen.

A commercial software program (KyPlot, KyensLab Inc., Tokyo, Japan) was used for statistical testing. The Wilcoxon signed rank test for paired data was used to statistically compare the differences in the elastic modulus and corneal thickness between specimens with and without intact epithelium. Differences with a *P* value of 0.05 or less were considered statistically significant. The statistical test was calculated (using GPower, ver 3.1.2) to have 81% power (SD: 30%; effect size: 1.25; sample size: 6) to determine differences ≥0.95 MPa and ≥33 µm in the Young's modulus and corneal thickness between specimens with and without intact epithelium respectively.

## Results

During the experiment, the room temperature and humidity of the moist chamber were 30.8±0.9°C and 74±5%, respectively. There were no differences in age between donor eyes with (mean: 66.00±4.58 years; range 62–71 years) and without intact epithelium (mean: 66.33±3.98 years; range 62–70 years).

### 3.1 Accuracy and repeatability of the procedure

The experimental error of the Pentacam was estimated to be 0.06 mm for radius of curvature, with a percent error of 0.8% with respect to the nominal values of the reference cornea. The surface fitting was highly accurate in all specimens. The mean least square error between the fitting model and the corneal elevation data exported from Pentacam were lower than 1^−28^ and 1^−35^ for the anterior and posterior cornea respectively. There were no differences in accuracy of the model between samples with or without intact corneal epithelium.

Under loading, the repeatability of measurements was 0.03 mm (0.4%), 0.29 mm (5.1%) and 8.02 µm (1.0%) for the anterior and posterior corneal curvature and the corneal thickness respectively. The 95% CI was between 0.02 and 0.04 mm, 0.20 and 0.42 mm, 5.41 and 10.72 µm respectively. Under unloading, the repeatability of measurements was 0.01 mm (0.2%), 0.15 mm (2.5%) and 8.04 µm (1.1%) for the anterior and posterior corneal curvature and the corneal thickness respectively. The 95% CI was between 0.01 and 0.02 mm, 0.10 and 0.20 mm, 5.36 and 10.71 µm respectively.

### 3.2 Cyclic regimen

**Figure 3:**
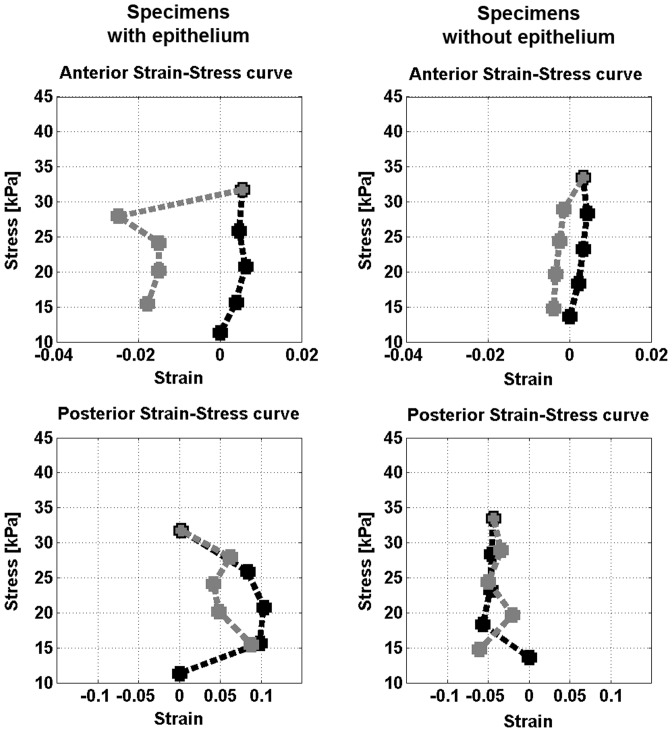
Stress-strain curves of the anterior and posterior cornea. Left column) Average stress-strain curves of the anterior and posterior cornea in specimens with intact epithelium. Right column) Average stress-strain curves of the anterior and posterior cornea in specimens without epithelium. In both plots, the black line represents the loading phase and the grey line the unloading phase of the *cyclic* regimen. The *x*-axis is shifted to start at 0. The anterior cornea of specimens with intact corneal epithelium showed higher strain than de-epithelialized tissues. Changes of epithelial thickness during testing contributed probably to estimate different anterior-side *E* values between specimens with and without epithelium. In unloading, negative strain indicates that the anterior cornea becomes steeper. The posterior cornea showed higher strain than the anterior cornea in all specimens. Although not a direct measure of hysteresis, the area inside the loading and unloading curves relates to the viscoelastic behaviour of the corneal tissue.

The anterior cornea showed smaller strain to *cyclic* stress application than the posterior cornea in all specimens. In specimens with intact epithelium, the average Young's modulus of the anterior and posterior cornea was 2.28±0.87 MPa and 0.21±0.09 MPa respectively. In specimens without epithelium, it was 3.30±0.90 MPa and 0.17±0.06 MPa respectively. The differences in *E* values between specimens with and without intact epithelium were statistically significant (P = 0.05). The average stress-strain curves of both specimens with and without intact epithelium are plotted in Figure 3. At the end of *cyclic* regimen, all specimens tended to recover their initial shape, however showing small residual visco-plastic deformation of both the anterior (<0.02) and the posterior cornea (<0.08), except for one sample (eye n. 060) that fully recovered its initial shapes.

**Figure 4:**
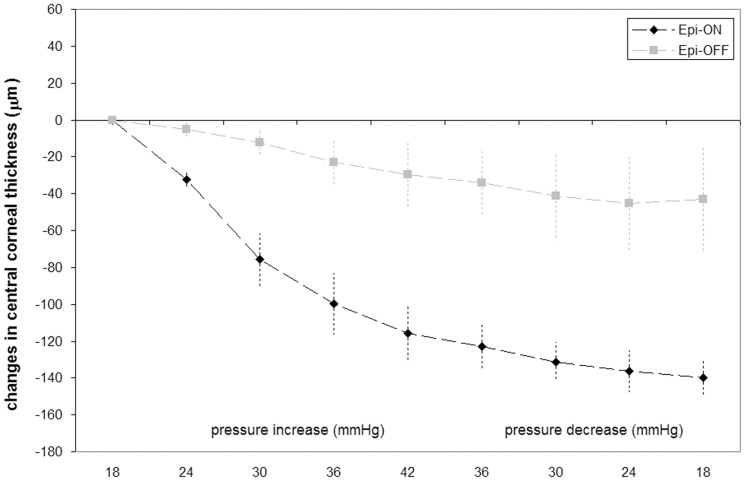
Central corneal thickness changes during *cyclic* regimen. Average changes of central corneal thickness (µm) with varying IOP pressure level in specimens with (black diamonds; Epi-ON) and without (grey squares; Epi-OFF) intact epithelium. Symbols represent average data across eye globes and error bars represent standard deviation.

The central corneal thickness (CCT) decreased during the *cyclic* regimen in all specimens, especially in those with intact epithelium (P<0.001), as shown in Figure 4. The CCT thinned of 139±11 µm (from 720- to 581-µm) and 43±26 µm (from 677- to 635-µm) in specimens with and without corneal epithelium respectively.

### 3.3 Creep regimen

**Figure 5:**
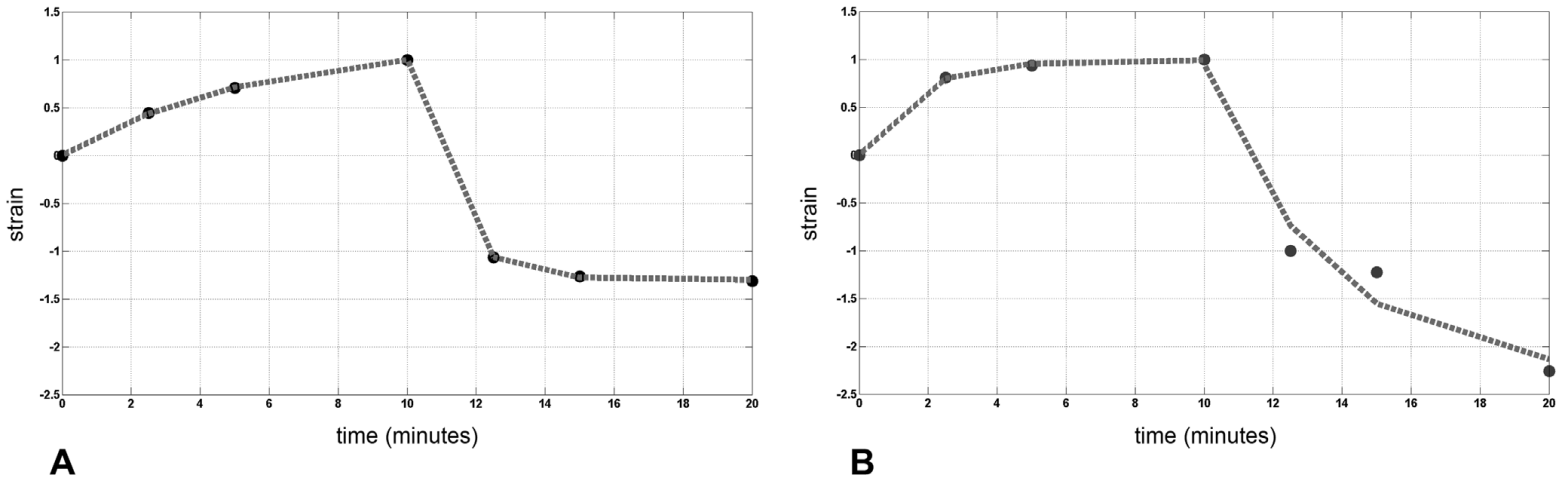
Strain response of the anterior cornea to *creep* regimen. Results obtained by fitting (grey dotted curve) the average experimental data (black dots) obtained from *creep* regimen of the anterior cornea in specimens A) with and B) without intact epithelium respectively. A) After sudden stress increase, the corneas with intact epithelium gradually strained to some amount. An immediate elastic recovery was found as soon as the stress was released. The relaxation times were τ_load_ = 5.50 minutes and τ_unload_ = 1.10 minutes in loading and unloading stress holds respectively. B) The specimens without intact epithelium showed an immediate strain after stress application and a slow recovery after stress release. The relaxation times were τ_load_ = 1.51 minutes and τ_unload_ = 4.69 minutes in loading and unloading stress holds respectively. At the end of *creep* regimen, a permanent, negative, strain remains, related to corneal steepening, both in specimens with and without corneal epithelium. In *creep* test, the magnitude of the permanent deformation depends on length of time, amount of stress applied, and environment conditions (temperature and humidity).

**Figure 6:**
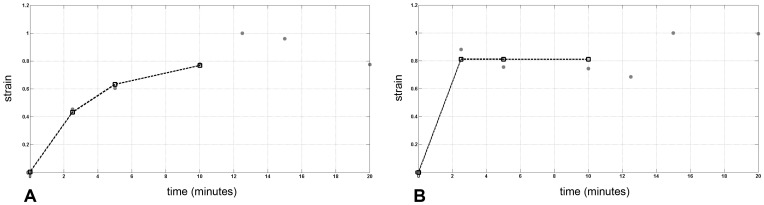
Strain response of the posterior cornea to *creep* regimen. Results obtained by fitting (black dotted curve) the average experimental data (grey dots) obtained from *creep* regimen of the posterior cornea in specimens A) with and B) without intact epithelium respectively. The modified Zener model fitted the loading creep curve of the posterior cornea. The relaxation times were τ*_load_* = 3.26 minutes and τ*_load_* = 0.09 minutes in specimens with and without epithelium respectively. After sudden stress release, the posterior cornea did not show any elastic recovery both in specimens with and without epithelium, and the model was unable to fit data. The response of the posterior cornea to sudden stress release appeared to be mostly dependent on the viscous component.

After sudden IOP increase and holding the IOP stable at 42 mmHg for 10 minutes, the anterior cornea showed a gradually increasing strain, then reaching a steady state value. After sudden IOP decrease and holding the IOP stable at 18 mmHg for 10 minutes, an immediate elastic recovery was found, followed by a period of recovery with a permanent deformation. The strain tended to a negative strain, i.e., the anterior surface became steeper than before *creep* test. The strain response of the anterior cornea to *creep* testing showed significant differences between specimens with and wihtout intact epithelium, as shown in Figure 5. After sudden stress application, the anterior cornea of specimens with epithelium took longer time (τ_load_ = 5.50 minutes; ***C***_∞load_ = 1.19 MPa^−1^) to relax to its strained steady state in comparison with the corneal tissues without epithelium (τ_load_ = 1.51 minutes; ***C***_∞load_ = 0.96 MPa^−1^). After sudden stress release, the specimens with epithelium (τ_unload_ = 1.10 minutes; ***C***_∞unload_ = −1.30 MPa^−1^) recovered corneal strain faster than specimens without epithelium (τ_unload_ = 4.69 minutes; ***C***_∞unload_ = −2.30 MPa^−1^). The posterior cornea of specimens with epithelium showed slower *creep* response to sudden stress application (*τ_load_* = 3.26 minutes; ***C***_∞load_ = 0.89 MPa^−1^) than specimens without epithelium (*τ_load_* = 0.09 minutes; ***C***_∞load_ = 0.70 MPa^−1^). After sudden stress release, no immediate elastic recovery of the posterior cornea was found in five of six specimens (2 specimens with epithelium and 3 without epithelium) and the model was unable to fit the strain response to the unloading creep curve (Figure 6). A permanent strain was found immediately after stress release.

After sudden loading and holding constant the pressure at 42 mmHg for 10 minutes, the CCT decreased by additional −6 µm and −8 µm in specimens with and without epithelium respectively. At the end of *creep* testing (i.e., 10 minutes after sudden unloading and holding constant the IOP at 18 mmHg), a total average decrease of CCT by −28 µm and −26 µm was found in specimens with and without epithelium respectively.

## Discussion

The traditional method used to test the corneal tissue biomechanics, i.e., the *tensile strip extensiometry* [15]–[19], involves applying a tensile force to strips of cornea held between two grips. Corneal biomechanics has been also measured by inflating the cornea within an artificial anterior chamber using internal pressure as the stress and measuring the strain by means of marks on the cornea (*bulge testing method*) [20]–[25]. Several factors limit the biomechanical measurements of excised donor human corneas, such as the corneal structure is disrupted, excessive swelling or dehydration of the tissue [26] and crucial constraints are ignored (meridional and thickness differences, boundary conditions), which can affect the viscoelastic response of the corneal tissue.

Inflation testing of the whole eye globe can be considered preferable for biomechanical testing of corneal tissue [27] because the procedure is minimally invasive, the native boundary conditions of the cornea are preserved and the applied stress is generated by IOP. In addition, the cornea can be analyzed with respect to its correct vertical/horizontal orientation, corneal tomography allows detailed characterization of the corneal surfaces with micrometric resolution, data may be easily exported for analysis and the results can be readily translated to clinic. In this study, we also monitored the humidity and temperature of the environment that may greatly influence corneal biomechanics.

In this work, we performed inflation testing on donor human eye globes in a controlled environment in order to investigate the anterior and posterior corneal response to *cyclic* and *creep* mechanical tests using commercial Scheimpflug device. We evaluated the strain response of the human cornea to physiologic IOP levels (18–42 mmHg) [28] generating a few assumptions on the corneal behaviour. A 27–60 mmHg pressure range has been previously shown to produce small viscoelastic deformations and a nearly linear pressure-deformation response [21], which suggested that, for physiological pressure ranges, the cornea may be reasonably approximated as a linear viscoelastic material. At this stage of our work, we were mainly interested to develop a reliable procedure to test biomechanics of the cornea *in situ*, rather than developing accurate model of corneal behaviour. Here, we used a model to estimate corneal strain to *cyclic* stress application including the assumptions of spherical topography and material homogeneity [29]–[32]. In addition, the mechanical properties of the sclera were not considered to contribute to the response of the cornea to IOP changes within physiological range. The model used to estimate the strain response to *creep* regimen was based on a modified Zener model [13] and was developed to describe the permanent strain that may be left in the corneal tissue in a constant-stress mechanical regimen [21].

The Young's modulus of the anterior cornea was consistently higher than the posterior cornea both in specimens with and without intact epithelium. The result is in agreement with previous work evaluating the reliability of Brillouin imaging to assess the biomechanical properties of bovine corneas in situ [33]. Brillouin imaging was able to visualize the spatially heterogeneous biomechanical properties of the cornea, while Scheimpflug imaging provided information on the local tissue mechanical properties; the anterior and posterior *E* values (bulk) may reflect a convolution of the properties of the most anterior and posterior stroma respectively. The result is also consistent with conventional mechanical measurements and knowledge on the differences in the stromal microstructure responsible for the mechanical performance of the tissue [11], [24], [25], [34]–[48]. The anterior stroma holds the main mechanical strength of the cornea and is responsible to maintain its shape and curvature. Anisotropy in stromal architecture results in mechanical anisotropy [34]–[43]. The anterior stroma is less hydrated than the posterior stroma with the anterior lamellae that are thinner, more densely packed and interwoven than the posterior lamellae. The stromal lamellae show increased order and width with stromal depth [44], [45]. Interlamellar cross-links are preferentially distributed in the anterior one third of the stroma. The deeper stroma has been found to have less strength and less resistance to swelling compared to anterior stromal layers [46], [47]. The anisotropic hydration of the stroma, increasing from the anterior to the posterior layers, was related to the distribution of different types of proteoglycans across the stroma [48]. Overall, the anisotropic stromal microstructure results in high stiffness in the front-back direction and our experimental procedure permitted to investigate this specific corneal property avoiding tissue excision [43].

Stress-strain curves of all specimens showed a hysteresis loop in a loading and unloading cycle, thus demonstrating the viscoelastic response of the cornea to *cyclic* mechanical testing. After both *cyclic* and *creep* regimens, a residual plastic negative deformation was found, that has been related to corneal steepening and tissue thinning [49]. The modified Zener model was able to completely fit the creep response of the anterior cornea to constant stress mechanical regimen of whole eye globes; on the other hand, it was unable to fit the strain response of the posterior cornea to sudden stress release from 42 to 18 mmHg, due to lack of immediate elastic recovery. The time dependent strain of the posterior cornea can be influenced by various factors, which include the amount and length of time of stress applied, the temperature and relative humidity, the stromal hydration state and the type and quantity of stromal proteoglycans.

Adequate control on tissue thickness before and during biomechanical experiments has been shown to provide predictive results on the corneal behaviour [50], [51]. Before commencing the experiment, donor eyes were kept in 15% dextran enriched storage solution at room temperature for 30 minutes and thereafter they were submitted to 15 minutes of *pre-stressing*. This procedure allowed us to use corneal tissues with similar thickness, ranging from 732 to 692 µm and from 698 to 645 µm in specimens with and without epithelium respectively, thus minimizing the influence of a different stromal hydration on the measurement of the elastic and viscoelastic behaviour of the cornea, as previously discussed [10], [24], [26], [50], [51], [52]. The presence of epithelium contributed to the differences (on average, 43 µm) in whole corneal thickness between specimens; Scheimpflug imaging was, however, unable to resolve reliably whether these differences were entirely due to the epithelial thickness. On average, central corneal thickness was found to decrease during mechanical testing, consistent with previous work [10]. The relative corneal thinning during mechanical testing was 20±4% and 11±5% in specimens with and without epithelium, showing consistency between specimens. A part of tissue deswelling was likely related to excessive post-mortem stromal fluid in the deep stroma. In addition, the epithelium may have been thinned during experiment. Maximizing storage and pre-stressing protocols of donors may further improve mechanical testing of intact eye globes [15], [26], [51], [52].

To understand the epithelial contribution to deformation mapping of the cornea, we evaluated the biomechanical response from ocular tissues with and without intact corneal epithelium. In inflation testing of corneal buttons, Elsheikh et al. [53] estimated the epithelium as having the 10–14% of the stiffness of a stromal layer of the same thickness. Using a ultrasound corneal elastometry, Dupps et al. [54] found substantial differences in stiffness after epithelial debridement with an average corneal stiffness increase by 20%. Thus, it is in general expected that the epithelium does not contribute significantly to the anterior corneal stiffness. In this work, the *E* value of the anterior cornea of specimens with intact epithelium was on average 30% lower than in specimens without epithelium. The presence of epithelium and differences in de-swelling during mechanical testing may have contributed to estimation of different anterior-side *E* values between specimens with and without epithelium. The *creep* testing demonstrated specific differences in both the immediate elastic and the retard visco-elastic responses between specimens with or without intact epithelium, likely related to the different time-dependent anterior-side response between specimens [15], [18], [55]. Overall, caution should be taken when comparing data between specimens with and without intact corneal epithelium in inflation experiment of eye globes [56]. Accurate modeling of the anisotropic properties of the cornea should be developed for accurate description of its biomechanical behaviour. In addition, it is recommended to clarify the environment condition and the type and extent of the stress applied to derive the creep viscoelastic behaviour of the human corneal tissue. Subjecting the corneal tissue to different stress levels and rate, longer exposure time and different temperature and humidity may significantly change the results of *creep* testing, because of variation in the stromal microstructure related to collagen/matrix re-arrangement [55]. Considerable corneal stiffening has been also shown with aging [57]. The increase in stiffness have been related to the additional non-enzymatic cross-linking of the stromal proteins. In this study, we included only eyes with the same range of donor ages (62–71 years) in order to avoid this type of bias.

Precise knowledge of shape is essential in understanding any mechanical system, and the corneal tomography is an accurate method to represent the corneal shape and information can be directly translated to clinical practice. Reliability of Pentacam measurements (accuracy of 0.06 mm with respect to the nominal values of a reference cornea) was in agreement with previous work [1], [58], [59]. The repeatability of curvature and thickness measurements was very high. The Pentacam instrument has been also demonstrated to consistently estimate anterior and posterior corneal deformation by intraocular pressure changes in porcine and human eyes that had undergone corneal cross-linking [10], [56].

In conclusion, the experimental procedure was shown to be reliable to test the viscoelastic properties of the intact human corneal tissue. Computerized tomography measurements acquired in inflation testing of eye globes can be valuable to ascertain the mechanical response of the human cornea *in situ* and better translate the results of innovative corneal treatments to clinic [1], [2].
